# Why Does Parental Divorce Lower Children’s Educational Attainment? A Causal Mediation Analysis

**DOI:** 10.15195/v6.a11

**Published:** 2019-04-16

**Authors:** Jennie E. Brand, Ravaris Moore, Xi Song, Yu Xie

**Affiliations:** a Departments of Sociology and Statistics, California Center for Population Research; and Center for Social Statistics.University of California, Los Angeles; b Department of Sociology, Loyola Marymount University; c Department of Sociology, University of Chicago; d Department of Sociology, Princeton University

**Keywords:** parental divorce, educational attainment, family income, psychosocial skills, causal mediation analysis

## Abstract

Mechanisms explaining the negative effects of parental divorce on children’s attainment have long been conjectured and assessed. Yet few studies of parental divorce have carefully attended to the assumptions and methods necessary to estimate causal mediation effects. Applying a causal framework to linked U.S. panel data, we assess the degree to which parental divorce limits children’s education among whites and nonwhites and whether observed lower levels of educational attainment are explained by postdivorce family conditions and children’s skills. Our analyses yield three key findings. First, the negative effect of divorce on educational attainment, particularly college, is substantial for white children; by contrast, divorce does not lower the educational attainment of nonwhite children. Second, declines in family income explain as much as one- to two-thirds of the negative effect of parental divorce on white children’s education. Family instability also helps explain the effect, particularly when divorce occurs in early childhood. Children’s psychosocial skills explain about one-fifth of the effect, whereas children’s cognitive skills play a minimal role. Third, among nonwhites, the minimal total effect on education is explained by the offsetting influence of postdivorce declines in family income and stability alongside increases in children’s psychosocial and cognitive skills.

PARENTAL divorce adversely affects a variety of children’s outcomes, including educational attainment (see McLanahan, Tach, and Schneider [2013] for a review). Children whose parents divorce are, on average, less likely to complete high school and attend and complete college. Mechanisms explaining the negative effects of parental divorce have long been conjectured and assessed. Sociologists have, unsurprisingly, suggested that a decline in family income is a central mechanism in the association between parental divorce and children’s educational attainment (Thomson and McLanahan 2012; Thomson, Hanson, and McLanahan 1994). With the loss of a parent in the household, typically fathers, mothers generally have fewer economic resources. It is well known that resource reduction negatively impacts children’s education, especially the ability to attend and complete college. Family instability (i.e., the number of transitions between remarriage, further divorce, cohabitation, and union dissolution) offers another plausible explanation. Relationship transitions occur more frequently following parental divorce, and such instability disrupts children’s lives and their schooling (Lee and McLanahan 2015; Sweeney 2010).

Scholars have also attended to the degree to which children’s skills—both cognitive and noncognitive—explain the lower level of educational attainment of children whose parents divorce. Some prior research treats these two components of children’s skills as parallel mechanisms that decline in response to family disruption (e.g., Kim 2011). Yet such a view obscures the way in which such skills develop over childhood. Although cognitive skills become relatively stable by early childhood, noncognitive skills, such as emotional and behavioral wellbeing, evolve and change throughout childhood and thus may change in response to disruptive family events (Borghans et al. 2008; Cunha and Heckman 2009; Hsin and Xie 2016; Roberts, Wood, and Caspi 2008). In other words, although both skills play an important role in children’s educational success (Sewell, Haller, and Portes 1969), their development follows independent trajectories (Cunha and Heckman 2009; Duncan and Magnuson 2011; Lleras 2008). The influence of parental divorce on children’s education via skill formation may depend on the type of skills and the stage of skill development when divorce occurs. Yet little is known about the relative explanatory power of children’s skills, both in comparison to one another and with respect to key explanatory factors such as family income and instability.

Although family scholars frequently emphasize the importance of these mechanisms, few studies of parental divorce have carefully attended to the assumptions and methods necessary to estimate causal mediation effects. Moreover, some children have large effects of divorce, whereas others have modest or even absent effects. Explaining the impact of divorce depends, of course, on whether such an effect exists for some subpopulations. One principal axis of variation is race: Studies of parental divorce have consistently documented a stronger association between parental divorce and children’s education for white than for nonwhite children (Amato 2001; McLanahan and Sandefur 1994). If we observe variation in total effects of parental divorce for white and nonwhite children, we should similarly attend to variation in mediating effects.

Although prior research has argued for the importance of family conditions and children’s skills in explaining the impact of parental divorce on children’s education, it has not adopted a causal mediation framework to assess the strength of these explanatory mechanisms. Using linked data from the National Longitudinal Survey of Youth (NLSY) and National Longitudinal Survey Child-Mother file (NLSCM), we assess the total and mediating effects of parental divorce on children’s educational attainment using a causal framework. We formally define mediation effects and outline assumptions necessary to maintain causal interpretations. We conduct sensitivity analyses based on assumptions about unobserved confounders and develop bounds for our estimates of the direct and mediating effects based on simulations. Using this approach, we quantify the relative strength and robustness of several key explanations for how divorce impacts children.

Our analyses yield three main findings. First, we confirm prior research showing that the effects of parental divorce on children’s education are larger for white children than nonwhite children. Indeed, after a rich set of potential confounders are considered, we find no negative effect of parental divorce on nonwhite children’s education. Second, family income and family instability mediate the negative effect of divorce among white children, explaining roughly one- to two-thirds or more of the effect. We also find that psychosocial skills mediate the effect for white children, whereas cognitive skills play no role in this process even if divorce occurred in early childhood. Children’s psychosocial skills play a relatively smaller role than family income and family instability, explaining about one-tenth to one-fifth of the effect. Third, we find that nonwhite children do not have significant declines in their education because declines in family income and stability are offset by increases in cognitive and psychosocial skills, especially in early childhood.

## Background

### Parental Divorce and Children’s Educational Attainment

U.S. families have changed dramatically since the mid-twentieth century. Between about 1950 and 1980, divorce rates more than doubled. Only one-quarter of marriages that began in the 1950s ended in divorce, whereas roughly half of all marital unions beginning in the 1970s eventually dissolved. The increasing incidence of divorce seemingly leveled off or declined after 1980 (Amato 2010; Rotz 2015; Stevenson and Wolfers 2007; yet, see Kennedy and Ruggles 2013). Still, since the 1980s, roughly half of children experience a parental divorce before they reach adulthood (Amato 2000; Fagan and Rector 2000). Family disruption is more likely to occur among low-income and black families (Amato 2010). In addition to socioeconomic and demographic factors, marital and fertility history, marital homogamy, relationship quality, traditional family values, and the circumstances surrounding a child’s birth predict marriage survival (Amato, Loomis, and Booth 1995; Kim 2011).

As the incidence of parental divorce increased, at least throughout the 1970s, the social stigma associated with such disruption lessened. Nevertheless, the negative consequences for children experiencing family disruption have persisted (Amato 2001). Substantial literature links parental divorce to lower levels of children’s educational attainment, particularly high school completion (see McLanahan et al. 2013).^1^ Scholars studying the causal effects of parental divorce on children have primarily relied on observational data, as divorce is a social phenomenon not subject to experimental manipulation. However, divorced families systematically differ from intact families, presumably in both observed and unobserved ways. Prior research on parental divorce has adopted a range of methods in an attempt to address concerns over selection into divorce (e.g., matching models, lagged dependent-variable models, individual and sibling fixed-effects models, and instrumental variable models), adding credibility to key findings regarding the negative effects of parental divorce on children’s attainment.

Several leading scholars of family instability have argued that researchers should attend less to the average effect of divorce and more to the factors that produce variability in children’s responses to divorce (e.g., Amato 2010; McLanahan et al. 2013). Research has shown that parental divorce has stronger effects on white children than on nonwhite children (Amato 2001; Lee and McLanahan 2015; McLanahan and Sandefur 1994; Wu and Thomson 2001). Amato and Keith (1991) and Amato (2001) in meta-analyses, for example, found that the impact of parental divorce on white children was nearly twice that of black children. Other findings suggest larger effects for children with more educated parents than children of less educated parents (Bernardi and Boertien 2016; Bernardi and Radl 2014; Martin 2012) and larger effects among children with a low propensity than those with a high propensity for parental divorce (Brand et al. [*forthcoming*]). We expect that exposure to a range of socioeconomic adversities among racial and ethnic minority children, like those among children with low socioeconomic status and children likely to experience family instability, renders the impact of any particular adverse event more normative and less severe (Brand and Simon Thomas 2014). In other words, nonwhite children have relatively low levels of educational attainment generally, and these levels do not substantially differ according to whether their parents remain married.

### Mechanisms That Account for the Relationship between Parental Divorce and Children’s Education

A causal mediation analysis provides estimates for the amount and proportion of the effects of parental divorce that are transmitted through various mechanisms. For a mechanism to mediate divorce effects on children’s education, it must satisfy two conditions: (1) The mechanism must be influenced by parental divorce, and (2) the mechanism must influence children’s educational outcomes. Moreover, to meaningfully assess the proportion of the divorce effect that is mediated by a mechanism, there must be an effect of divorce to explain. If parental divorce does not affect the education of some children, it is nevertheless useful to differentiate between whether different mechanisms offset one another to produce a null result.

The divorce literature has focused on several plausible mechanisms that reasonably satisfy the two conditions described above. Our investigation is not meant to be an exhaustive accounting of all possible mechanisms but a comparison of key indicators of family conditions and children’s skills. Family income is a central mechanism linking parental divorce to child wellbeing. Divorce is associated with a decline in family income (condition 1), and decades of social science research demonstrates that family income plays a major role in children’s education (condition 2) (e.g., Crosnoe and Cavanagh 2010; Duncan et al. 1998; Lee and McLanahan 2015; McLanahan and Sandefur 1994). In addition to the substantial impact on home, neighborhood, and school environments; health and emotional wellbeing; and procuring educational goods and resources, family income is directly associated with parents’ ability to pay the increasingly high price of college (Goldrick-Rab 2016).^2^ Prior research suggests that differences in family economic resources account for a substantial share of the differences in child outcomes across family types (McLanahan and Sandefur 1994; Thomson and McLanahan 2012; Thomson et al. 1994). Indeed, McLanahan and Sandefur (1994) argue that family income is the single most important mediator, explaining roughly half of the effect of a parental divorce on education. Their estimate is based on divorce that occurred during adolescence on the likelihood of dropping out of high school. Although white families, who on average are more advantaged, are likely to have higher levels of economic resources than nonwhite families, resource loss as a result of divorce may be more pronounced in the former than in the latter, leading to worse outcomes for children’s education (Bernardi and Boertien 2016). For a greater proportion of nonwhite families, income may have already been below the threshold of investment in higher education prior to resource decline due to divorce.

Family instability, in the form of transitions in household composition and family relationships, is a second key mechanism implicated in the literature on parental divorce. Family instability satisfies the two conditions needed for mediation. Relationship transitions are more likely following a parental divorce (condition 1) and are associated with high levels of parenting stress and lower-quality parent-child relationships, leading to lower attainment among children (condition 2) (Beck et al. 2010; Cavanagh and Huston 2006; Halpern-Meekin and Turney 2016; Lee and McLanahan 2015; Osborne and McLanahan 2007; Thomson and McLanahan 2012; Waldfogel, Craigie, and Brooks-Gunn 2010; Wu and Thomson 2001). White families may experience fewer subsequent transitions than nonwhite families who have a high likelihood of disruption. However, as with declines in family income, increased instability may play a more consequential role in limiting children’s education among families unprepared for disruption.

We also consider children’s psychosocial and cognitive skills as mechanisms by which family disruption limits education. Psychosocial skills (also termed “noncognitive skills,” “socioemotional skills,” and “personality traits”) encompass a broad class of attitudes and behaviors that are correlated with but distinct from cognitive ability, such as emotional stability, self-esteem, mastery, conscientiousness, locus of control, and behavior (Borghans et al. 2008; Claessens, Duncan, and Engel 2009; Heckman, Stixrud, and Urzua 2006; Lleras 2008; Rosenbaum 2001). Psychosocial skills evolve and change from early childhood through adolescence, allowing the family environment to play a significant role in shaping development (Hsin and Xie 2016; Roberts et al. 2008).^3^ We thus expect condition 1 to be satisfied for psychosocial skills. Regarding condition 2, the status-attainment tradition indicated a role for expectations and aspirations (Sewell et al. 1969), and scholars recognize the critical role of a broad class of psychosocial skills in influencing children’s academic achievement and educational attainment (Cunha and Heckman 2009; DiPrete and Jennings 2012; Duckworth and Seligman 2005; Duncan and Magnuson 2011; Hsin and Xie 2016; Jackson 2006; Lleras 2008; Rosenbaum 2001; Wolfe and Johnson 1995) even among individuals who share the same family background and cognitive ability (Heckman and Rubinstein 2001; Heckman et al. 2006).

Given evidence in support of both conditions 1 and 2, we hypothesize that psychosocial skills mediate the effect of parental divorce on children’s education. We presume that various additional factors, such as parents’ psychological wellbeing, parenting style, and family relations, influence children’s educational attainment primarily by way of their impact on children’s psychosocial skills (Cheadle and Amato 2010; Meadows, McLanahan, and Brooks-Gunn 2007; Turney 2011). The marginal effect of psychosocial decline may nevertheless differ across families. Psychosocial decline may be more pronounced among children unaccustomed to socioeconomic disadvantage and disruption, who experience a greater psychological shock from parental divorce. Parental divorce may, by contrast, not lead to decline among children who have grown accustomed to adverse events in their lives via already higher levels of socioeconomic instability and family conflict (Brand et al. [*forthcoming*]). In fact, the dissolution of the union may even offer some psychological relief from a high-conflict environment (Amato 2010; Thomson and McLanahan 2012). We expect more white children to compose the former and more nonwhite children to compose the latter scenarios.

Cognitive ability has played a central role in models of status attainment (Sewell et al. 1969) and human capital development (Becker 1993), and such skills impact educational outcomes and satisfy condition 2. Condition 1, however, that parental divorce impacts cognitive skills, must also be satisfied for such skills to mediate the relationship between divorce and children’s education. Whereas some research treats children’s psychosocial and cognitive skills as symmetrical (e.g., Kim 2011), we maintain that the developmental literature points to important asymmetry in the acquisition of such skills. Cognitive skills undergo rapid development in early childhood and, in contrast to psychosocial skills, gradually stabilize thereafter (Borghans et al. 2008; Cunha and Heckman 2009). Hence, if we observe an impact of parental divorce on children’s cognitive skills (e.g., Kim 2011), at least beyond the early childhood years, it is likely influenced by the impact of psychosocial mediators on test scores used to measure cognition. The evidence for the effects of parental divorce on cognitive assessments in math, verbal, and general test scores is mixed, with studies adopting more stringent tests for causal associations suggesting little or no effect (Aughinbaugh, Pierret, and Rothstein 2005; Cherlin et al. 1991; Lee and McLanahan 2015; Morrison and Cherlin 1995; Sun 2001; Sun and Li 2002). It is thus important to consider developmental periods when assessing mechanisms accounting for the impact of disruptive family events on children’s attainment. Some work suggests that early childhood may be especially sensitive to family disruption (McLanahan et al. 2013). We analyze the entirety of individuals’ childhoods, dividing their developmental stages into early childhood, middle childhood, and adolescence, and compare children’s educational outcomes according to age at the time of parental divorce.

## Analytical Approach

Three types of estimates contribute to our empirical results. First, total effects quantify the overall impact of parental divorce on children’s educational attainment. Second, mediating effects quantify the portion of the total effect transmitted through a given mediator, and the proportion mediated offers an estimate of the percentage of the total effect attributable to that mediator. Third, sensitivity analyses quantify how the total and mediating effects change in the presence of an unobserved cofounder. The following sections detail the causal framework underlying these estimates.

### Estimating Total Effects of Parental Divorce on Children’s Educational Attainment

For a focal child *i*, the total treatment effect (*TE*) of parental divorce is defined as the difference between the two potential outcomes in the treated (i.e., divorced parents) and untreated (i.e., nondivorced parents) states (*D* = 1, 0):

(1) $$TE_{i}=Y_{i}\left(\text{1}\right)-Y_{i}\left(0\right)$$

That is, we ask whether a child whose parents divorced had different outcomes than he or she otherwise would have had if his or her parents had not divorced. Given the impossibility of observing both treated and untreated outcomes for any individual, the individual-level causal effect is unidentifiable. With observational data, the researcher can estimate group-level causal effects under various assumptions. A key assumption is ignorability (i.e., the assumption that parental divorce is uncorrelated with unobserved factors that affect children’s outcomes). To guard against potential selection bias and improve confidence in the ignorability assumption, we condition the analyses on a rich set of observed characteristics (shown in Table 1), which is indeed a more extensive set than most prior analyses of marital disruption. Still, the ignorability assumption may not hold true, as parents may self-select into divorce because of unobserved factors.

Our analytical approach begins with the estimation of the propensity for parental divorce (*P* = *P* (*D*_*i*_ = 1 | *X*_*i*_)) based on observed covariates (*X*) using a logit regression model. Under the ignorability assumption, conditioning on the propensity score is as sufficient as conditioning on the full array of covariates *X* for the estimation of treatment effects (Rubin 1997). Departing from most previous research on parental divorce effects on children, our approach necessitates that we explicitly model parental divorce as a first step. We then estimate an average treatment effect conditional on the observed propensity for parental divorce:

(2) $$ATE_{p}=E(Y(1)-Y(0)|P=p).$$

*ATE*_*p*_ measures the total effect of parental divorce operating through all mediating pathways. We estimate a series of linear probability models of the effects of parental divorce on children’s high school completion, college attendance, and college completion:^4^

(3) $$Y_{i}=\alpha +\beta_{1}D_{i}+\beta_{2}P_{i}+\varepsilon_{i}.$$

For simplicity and ease of interpretation, we include the propensity score as a linear term in equation 2.

We also assess whether the total effects of parental divorce vary by race. We underscore that effect variability, as indicated by stratified analyses may result, at least partially, from differential unobserved selectivity (Xie, Brand, and Jann 2012; Zhou and Xie [*forthcoming*]). That is, white parents have a lower observed likelihood of divorce and may also have lower unobserved resistance to divorce (Brand et al. [*forthcoming*]). We also stratify analyses by child age when divorce occurs using the conceptual approach of Brand and Xie (2007). Divorce occurs in period *d* = *t*, where *t* is given by three age ranges (i.e., 0 to 5, 6 to 11, and 12 to 17). As a simplifying assumption, the analysis only considers a child’s first parental divorce event. Analogous to an event history setup, children at risk for experiencing parental divorce at time interval *t* have not experienced the event up to the baseline of *t*. The reference children include all those who have not experienced parental divorce up through time *t* and those who do and do not experience parental divorce at any time subsequent to *t*.

### Estimating Mediating Effects of Parental Divorce on Children’s Educational Attainment

**Figure 1:**
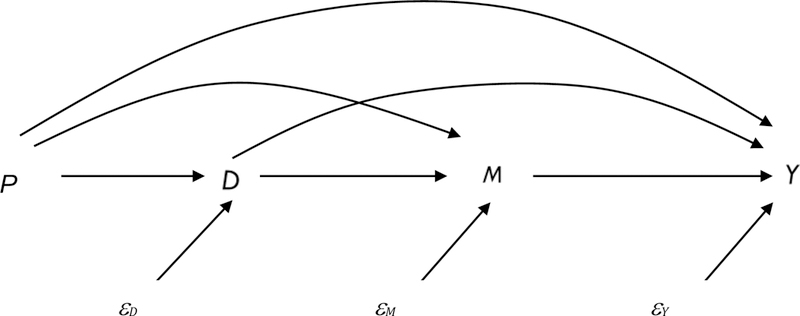
A causal framework based on a DAG. *P* = propensity for parental divorce; *D* = parental divorce; *M* = postdivorce mediators (children’s psychosocial skills, children’s cognitive skills, family income, and family instability); *Y* = children’s educational attainment (high school completion, college attendance, and college completion).

A causal mediation analysis is designed to assess mechanisms through which a treatment affects an outcome. Mediation methods using a potential outcomes framework have rapidly expanded in recent years (VanderWeele 2016). A potential outcomes approach provides a coherent framework clarifying the assumptions needed to estimate valid mediation effects (Hicks and Tingley 2011; Imai, Keele, and Tingley 2010; Imai, Keele, and Yamamoto 2010; Keele, Tingley, and Yamamoto 2015; Pearl 2001,^2009^; Robins and Greenland 1992; VanderWeele 2015,^2016^). The goal in causal mediation analyses is to decompose the total treatment effect (i.e., *ATE*_*p*_ in equation 2) into direct and mediating (or indirect) treatment effects. The mediating effect reflects one potential pathway through which the treatment produces the effect on the outcome of interest. Although it is infeasible to identify all indirect influences of parental divorce on children, we examine four mediators—family income, family instability, children’s psychosocial skills, and children’s cognitive skills—the roles of which have been widely implicated in the literature but the relative importance of which have not been rigorously tested. Figure 1 is a directed acyclic graph (DAG) that illustrates the relationship between the propensity for parental divorce (*P*), parental divorce (*D*), the mediators (*M*), and children’s educational attainment (*Y*).

Let *M*_*i*_ (*d*) denote the potential value of the mediator that would be realized under treatment status *D* = *d*. For example, *M*_*i*_ (*d*) may indicate child *i’*s postdivorce psychosocial skills that would have been observed had the child experienced a parental divorce (*D* = 1) or not (*D* = 0). Only the potential mediator that corresponds to the actual received treatment is observed. Let *Y*_*i*_ (*d, m*) represent the potential outcome that would result if the treatment and mediating variables equaled *d* and *m*, respectively, for *i*. For example, *Y*_*i*_ (1, 0.6) represents high school completion status for child *i* that would be observed if the child had experienced parental divorce and the psychosocial skills scale equaled 0.6 (the mean value for children of divorced parents). The observed *Y*_*i*_ (1, 0.6) is only one of many potential outcomes of *Y*_*i*_ (*d*, *M*_*i*_ (*d*)).

Using this notation, we define the total treatment effect for unit *i* as follows:

(4) $$TE_{i}=Y_{i}\left(1,M_{i}(1)\right)-Y_{i}\left(0,M_{i}(0)\right).$$

This is the same effect described in equation 1, yet equation 4 explicitly expresses the mediating mechanisms. We define the causal mediation effect of the treatment, also known as the natural indirect effect (*NIE*) (Pearl 2009), on the outcome through the mediating variable for unit *i* as follows:

(5) $$NIE_{i}=Y_{i}\left(d,M_{i}(1)\right)-Y_{i}\left(d,M_{i}(0)\right).$$

The indirect effect shows what change would occur to the outcome if the mediator changed from what would be observed when units are treated (*M*_*i*_ (1)) to what would be observed when units are untreated (*M*_*i*_ (0)) while holding the treatment status constant at *d*.^5^ This deactivates all pathways except for that operating through the focal mediator. For example, *Y*_*i*_(1, *M*_*i*_(1)) may represent college attendance for child *i* with divorced parents and the level of psychosocial skills after the parents divorced, and *Y*_*i*_ (1, *M*_*i*_(0)) may represent college attendance for the same child with divorced parents but with the level of psychosocial skills had the parents not divorced. The mediating effect in this example explains the degree to which parental divorce impacts college attendance by decreasing children’s psychosocial skills.^6^ To identify mediating effects, we estimate expected values of *TE*_*i*_ and *NIE*_*i*_ by adjusting for observed covariates under the assumption of sequential ignora-bility. That is, we assume no treatment-outcome confounding (as indicated above) as well as no treatment-mediator confounding or mediator-outcome confounding (VanderWeele 2016). We can then estimate the proportion of the total effect that is indirect:

(6) $$PM=\frac{NIE}{TE}.$$

Our mediation analysis proceeds as follows. First, we fit a regression predicting the mediator (*M*) that includes the treatment (*D*) and the propensity for treatment (*P*):

(7) $$M_{i}=\alpha_{m}+\beta_{m1}D_{i}+\beta_{m2}P_{i}+\varepsilon_{im}.$$

Second, we fit a regression predicting the outcome that includes the mediator, treatment, and relevant covariates:

(8) $$Y_{i}=\alpha_{y}+\beta_{y1}D_{i}+\beta_{y2}P_{i}+\beta_{y3}M_{i}+\varepsilon_{iy}.$$

We simulate model parameters in the mediator and outcome models from their sampling distributions. For each simulation, on the basis of the mediator model, we generate two sets of predicted mediator values for each unit: one when *D* =1 and one when *D* = 0. We use the outcome model to impute potential outcomes: first, the predicted outcome and the mediator value when *D* = 1 (from the previous step) and second, the predicted counterfactual outcome and the mediator when *D* = 0. The average causal mediation effect is obtained by averaging the differences between the predicted outcomes under the two values of the mediator across units. For example, we could generate the average difference in children’s college attendance across levels of psychosocial skills with and without experiencing parental divorce. We repeat the simulation 1,000 times to obtain estimates of uncertainty and statistical significance tests (see Appendix D in Imai et al. [2010] for technical details).

### Sensitivity Analyses for Total and Mediating Effects

An advantage of our formal causal mediation framework is that we conduct sensitivity analyses that allow us to determine when the total and mediating effects become insignificant in the presence of an unobserved confounder. That is, our sensitivity analyses quantify how the results obtained under the sequential ignorability assumption would change if that assumption were relaxed. A standard approach is the calculation of a bias factor (Arah 2017; Gangl 2013; VanderWeele 2015, 2016; VanderWeele and Arah 2011). The sensitivity of the estimated total effects to unobserved treatment-outcome confounding can be assessed by subtracting the bias factor from the point estimate and confidence interval of the treatment effect. For simplicity, let us consider an unobserved binary confounder (*U*). The bias term is equal to the product of two parameters:

(9) B=γλ,

where

(10) γ=E(Y|u=1,D=d,P=p)−E(Y|u=0,D=d,P=p)

and

(11) λ=P(u=1|D=1,P=p)−P(u=1|D=0,P=p).

That is, *γ* is the mean difference in children’s education associated with *u*, and *λ* is the mean difference in *u* between the children of divorced and nondivorced parents, both being conditional on the estimated propensity for divorce. We assess the sensitivity of the mediation effects to the assumption of unobserved mediator-outcome confounding with another bias term. The components of the bias term are analogous to those for the total effects except that they are now conditioned on the mediator. The bias term is, in this case, equal to the negation of the product of the two parameters, and we subtract this bias term from the mediation effect and the confidence interval.^7^ We conduct a series of sensitivity analyses by race and children’s age at which parental divorce occurs.

## Data

We use data from the NLSY, which is a nationally representative sample of 12,686 respondents who were 14 to 22 years old when first surveyed in 1979. These individuals were interviewed annually through 1994 and biennially thereafter. In 1986, the National Longitudinal Survey began a separate survey of the children of NLSY women, the NLSCM. Data have been collected every two years since 1986, with new sections being added in 1994 as children entered young adulthood. As of 2012, the 6,283 women of the NLSY were 47 to 54 years old and had given birth to about 11,500 children. Several prior studies have used data from the NLSY to investigate the impact of parental divorce (e.g., Aughinbaugh et al. 2005; Lang and Zargorsky 2001; Morrison and Cherlin 1995). We linked data on women from the NLSY with data on children from the NLSCM (*n* = 11,512 children; *n* = 4,931 mothers) and treat children as our units of analysis.

We constructed measures of whether and when a child (0 to 17 years old) experienced a parental divorce using NLSCM-provided month and year of birth for children and NLSY-provided marriage start and end dates for parents. We identified 8,319 children of 3,940 mothers who were born into marriage—that is, children at risk of experiencing parental divorce over childhood. This restriction allows for the examination of a relatively homogenous population of children. We then identified children who experienced parental divorce at or before age 17 and further restricted the sample to those who were at least 18 years old by 2012 (*n* = 7,258 children). About one-third of our sample *(n* = 2,420 children) experienced parental divorce throughout childhood. The average age of children at the time of divorce is 7 years old.

### Descriptive Statistics

Drawing on prior research on the determinants of divorce, we include a rich set of covariates shown in Table 1 to construct the propensity of parental divorce over childhood: family background characteristics (i.e., maternal race, nation of origin, residential location, religion, family structure and size, and household income of mothers during childhood); socioeconomic characteristics (i.e., maternal education, employment status, job conditions, delinquency, household income, poverty, and welfare status); cognitive and psychosocial skills (i.e., maternal scales for Rotter locus of control, Pearlin mastery, Rosenberg self-esteem, delinquency [based on 16 questions regarding stealing, gambling, fighting, and drugs], depressive symptoms [7-item Center for Epidemiologic Studies Depression scale (CESD)], body mass index, cognitive ability [Armed Services Vocational Aptitude Battery (ASVAB) test], and high school academic achievement [class rank and college preparatory program]); and family formation and wellbeing factors (i.e., maternal early sexual activity; beliefs about traditional family roles; age at the time of the child’s birth; prior marriages; time between marriage and first birth; desirability of the birth of a child^8^; child gender and birth weight; whether parents argue about chores, money, cheating, or religion; and whether parents match with respect to religion, race, and education). Missing values for the covariates were imputed on the basis of predivorce characteristics. We observe significant differences by parental divorce status for most of the indicators we include, suggesting greater socioeconomic disadvantage and lower family wellbeing among parents who divorce.^9^ The final column of Table 1 provides a balance test, which indicates that almost all the covariates are no longer significant predictors of parental divorce when adjusting for the propensity of divorce. Maternal family size, parental income, ability, and months between marriage and first birth are exceptions, yet adjusting for these in our models of the effects of parental divorce on children’s education has no substantive impact on our estimates.

Table 1 also describes the mediators and outcomes used in the main analyses. Measures of children’s educational attainment include high school completion by age 18, college attendance by age 19, and college completion by age 23. Measures of children’s skills include a scale of cognitive skills and a scale of psychosocial skills. The psychosocial skills scale is constructed using five indicators: the Pearlin Mastery Scale, the Rosenberg Self-Esteem Scale, the Ten-Item Personality Inventory (TIPI) Emotional Stability scale, the Behavioral Problem Index (BPI), and the CESD.^10^ These indicators were all measured when children were ages 15 and older, except indicators of behavioral problems, which were measured between ages 4 and 15. For both the cognitive and psychosocial skills scales, the selected items were standardized to have a zero mean and unit variance. We took the mean value of the standardized values to create a composite scale measure and then transformed each scale measure onto the (0,1) interval. The cognitive skills scale is constructed by averaging three Peabody Individual Achievement Test indicators: Reading Comprehension, Reading Recognition, and Math. Children are between ages 5 and 18 when tested.^11^ Relationship transitions or family instability include the number of times a transition occurs between the statuses of married, separated, remarried, widowed, and cohabitating and thus can have positive values for both divorced and nondivorced families.

All mediators were constructed as averages of the measures over the years subsequent to the parental divorce event. For example, if a child’s parents were divorced when the child was 7 years old, we averaged the values of the mediator between ages 8 and 17. Construction of mediators offers inherent measurement challenges due to nonrandom selection into divorce, the timing of divorce, and the expectation that mediators exhibit some degree of age dependency. To assess the degree to which differences in mediators explain effects on children’s educational attainment, we need to compare postdivorce mediator estimates for the divorce group to an analogous estimate for children who do not experience parental divorce. Yet whereas children whose parents divorce have an observable event time, children whose parents do not divorce have no analogous event time.^12^ To address this issue, we employ a method that matches children from the divorce group to children in the nondivorce group on the basis of their gender and propensity to experience parental divorce. After the match, we simulate age at divorce for the control group child as the observed age at divorce for the matched child in the treated group. We then average all measures taken after the divorce age (observed or simulated; up to age 17) to create a postdivorce mean.^13^ Appendix Table A in the online supplement shows that children whose parents divorced have lower levels of psychosocial and cognitive skills, lower family income, more frequent family transitions, and lower levels of educational attainment.

## Empirical Results

### Predicting Parental Divorce

We first model the probability that a child experiences parental divorce over the course of childhood (ages 0 to 17) as a function of the covariates described in Table 1. For the full model, pretreatment covariates correspond to those at the time of the child’s birth. Our age-specific analyses condition on time-invariant and time-varying covariates. As results from models predicting parental divorce are seldom presented in prior work on divorce effects on children, the literature has not established a widely accepted prediction model. Our model incorporates a rich set of theoretically informed covariates based on the literature on the determinants of divorce.^14^

Results reported in Appendix Table A in the online supplement show that mothers who themselves were raised in large families with fathers present during childhood are less likely to divorce. Mother’s self-esteem is negatively associated with the odds of divorce, and a high level of CESD-detected depressive symptoms is positively associated with the odds of divorce. High cognitive ability, self-mastery, and academic achievement in high school among mothers appear to be positively associated with divorce. Education and household income generally reduce the odds of divorce, whereas mothers’ employment, especially employment at a private company without flexible hours, increases odds of divorce. Family formation factors strongly influence the likelihood of divorce, with women adopting more traditional family practices (e.g., delayed sexual debut and no prior marriages) and attitudes being less likely to divorce. Relationship quality measures indicate that arguing about chores is positively associated with divorce, whereas arguing about money is negatively associated with divorce. Parents who differ in their educational attainment and who are of different races are more likely to divorce. Yet parents raised in different religions are less likely to divorce, perhaps reflecting strong selection into cross-religion marriages. In sum, with some notable exceptions, the likelihood of divorce generally declines with socioeconomic status and family wellbeing.

### Total Effects of Parental Divorce on Children’s Educational Attainment

We present linear probability model estimates of the total effects of parental divorce on children’s educational attainment in Table 2. The first column reports effect estimates for the full sample adjusted for the propensity of parental divorce and child age. We observe that divorce is associated with a 4-percent-lower probability of children’s high school completion, a 7-percent-lower probability of college attendance, and a 7-percent-lower probability of college completion. Holding the propensity for parental divorce at the median, we predict that among children whose parents stay married, about 81 percent complete high school, 56 percent attend college, and 23 percent complete college, whereas among children whose parents divorced, about 78 percent complete high school, 50 percent attend college, and 17 percent complete college.

In the latter two columns, we present effect estimates separately by race. We find sizable effects for white children: a 7-percent-lower level of high school completion (79 percent relative to 86 percent predicted value with the propensity held at the median), a 13-percent-lower level of college attendance (51 relative to 64 percent), and a 13-percent-lower level of college completion (19 relative to 32 percent). We find no significant effects for nonwhite children, with point estimates being near zero. That is, although predicted probabilities of each level of educational attainment are low for nonwhites (i.e., about 75 percent for high school completion, 46 percent for college attendance, and 13 percent for college completion), these levels do not differ between the children of divorced and nondivorced parents. We underscore that levels of educational attainment among nonwhite children of married parents are lower than those among white children of divorced parents.

In Table 3, we present estimates stratified by race and three developmental periods according to when parental divorce occurs: early childhood (ages 0 to 5), middle childhood (ages 6 to 11), and adolescence (ages 12 to 17). These models incorporate time-varying characteristics of families and thus also provide a potentially better adjustment for confounding variables. We find large effects for white children, with a similar impact for divorce occurring in early and middle childhood (i.e., about a 7-percent-lower level of high school completion, and a 9- to 12-percent-lower level of college attendance and completion). We observe a somewhat smaller effect for parental divorce that occurs in adolescence: a (marginally significant) 5-percent-lower level on high school completion and a 7-percent-lower level on college attendance and a 10-percent-lower level of college completion. By contrast, we again find no significant negative impact of parental divorce for nonwhite children across any age at the time of divorce and one marginally significant positive effect on college completion for parental divorce that occurs in early childhood (a 4-percent-higher level). Indeed, point estimates of parental divorce that occurs in early childhood among nonwhite children are all positive, though estimated imprecisely. Estimated effects of divorce that occurs in middle childhood and adolescence are negative, ranging from 1 to 4 percent, but are again imprecise.

### Mediating Effects of Parental Divorce on Children’s Educational Attainment

In Table 4, we report estimates of the mediating effects of children’s psychosocial skills, cognitive skills, family income, and family instability for white children by age when divorce occurs. As each mediator is assessed in turn, the proportion mediated does not sum to 100 percent for a given outcome. We only report the proportion mediated for precisely estimated total and mediating effects (significant at the 0.05 level at least). The mediating influence of family income is high for all levels of educational attainment (accounting for about 30 to 40 percent of the total effect) but particularly high for college completion among adolescents whose parents divorced (accounting for 67 percent of the effect). Family instability also explains a substantial portion of the effect of parental divorce on children’s education (anywhere from about 20 to 40 percent). Instability is particularly explanatory when divorce occurs in early childhood, leaving more time for a greater number of transitions to ensue. Divorce-induced changes in children’s psychosocial skills account for more than 20 percent of the total effect of divorce when divorce occurs in early childhood (and 25 percent on college completion) and 15 percent of the effect on college completion when divorce occurs in adolescence. We find no significant mediating effect of children’s cognitive skills, confirming our expectation that psychosocial, more so than cognitive, skills link parental divorce to lowered attainment among children. This is true even for parental divorce that occurs in early childhood, when such skills are more malleable.

We report results for mediating effects for nonwhites by age of divorce in Table 5. No total effects are precisely estimated for nonwhites, and thus, we do not report the proportion mediated. Yet the mediating effect estimates inform our understanding of the negligible divorce effects on nonwhite children’s education. We find that the negative mediating effects of family income and family instability are seemingly offset by positive mediating effects of psychosocial skills and cognitive skills. In fact, the positive mediating effect of cognitive skills and (marginally significant) effect of psychosocial skills in response to divorce that occurs in early childhood lead to (imprecise) positive total effects of divorce on educational attainment. Declines in family income ostensibly lead to the negative point estimates for divorce that occurs in adolescence among nonwhites despite some positive effects on children’s skills.

### Sensitivity Analysis for Total and Mediating Effects of Parental Divorce on Children’s Educational Attainment

In our preceding analyses, we invoked the sequential ignorability assumption. Whether this assumption is reasonable is a substantive rather than a methodological issue, which depends upon the quality of the exogenous covariates in capturing potential selection bias. We include an extensive set of covariates to predict divorce. Yet if there remain unobserved confounding variables that impact outcome variables as well as parental divorce or the proposed mediators, the sequential ignorability assumption would be violated and the total and mediation effects unidentified (Keele et al. 2015). We recognize that even with a rich set of pretreatment covariates, potential confounders may remain (e.g., unobserved paternal characteristics). We address the possibility of unobserved confounding for the total and mediating effects with a series of sensitivity analyses.

In sensitivity analyses for total effects, we assume that the unobserved con-founder is a binary variable and assess values from −40 to 40 percent for γ with values of −5 to −10 percent for λ. Very few characteristics of children of divorced and nondivorced parents differ by more than 5 to 10 percent. As the bias factor is the product of γ and λ, the effect reaches nonsignificance when the unobserved confounder has a strong effect on children’s education or a large difference between children of divorced and nondivorced parents. Suppose, for example, that fathers’ full-time employment status, unobserved in our data, enhances levels of education and is less common among fathers who get divorced (Killewald 2016). When λ equals −10 percent, we assume that the prevalence of fathers having been employed full time is 10 percent less in the divorced group than in the nondivorced group; when γ equals 20 percent, we assume that children whose fathers are employed full time have a 20 percent advantage in graduating from high school (or attending or completing college) over children whose fathers are not employed full time (all else held equal). As reported in Appendix Table B1 in the online supplement, we find that the total effect of divorce on high school completion, college attendance, and college completion for children whose parents divorced in early and middle childhood remains significant when λ is 5 percent and γ is 40 percent (an implausible value) and when λ is 10 percent and γ is 20 percent. The significant total effects of divorce on educational attainment reported in Table 3 are thus highly robust to unobserved confounding. The marginally significant effects for children whose parents divorce in adolescence on high school completion and college attendance are, as expected, more sensitive to unobserved confounding.

In sensitivity analyses for mediating effects of family income, instability, and psychosocial skills, we let 7 range from −20 to 20 percent and *λ* range from −5 to −10 percent.^15^ Suppose, for example, that fathers’ lack of financial contribution to college education is an unobserved confounder in the relationship between family income and children’s education, such that λ and γ are negative. That is, fathers not contributing to college costs is more prevalent among divorced families and decreases children’s education attainment. Omitting this confounder, we may overstate the mediating effect of family income. As reported in Appendix Table B2 in the online supplement, we observe that family income mediating effects remain significant with γ at 20 percent and λ at 10 percent for most outcomes (which is equivalent to values of γ at 40 percent and λ at 5 percent). Mediating effects of relationships transitions and children’s psychosocial skills generally remain significant with γ at 10 percent and λ at 10 percent but are, in some instances, reduced to nonsignificance with λ at −5 percent.

## Summary and Discussion

Children whose parents divorce have, on average, lower levels of educational attainment than children whose parents stay together. Yet not all children respond the same way to their parents divorcing. We find that parental divorce limits white children’s, but not nonwhite children’s, educational attainment. This finding supports prior research on racial differences in the impact of parental divorce. We note that the level of educational attainment among nonwhite children is fairly low—indeed lower than that of white children with divorced parents—and that the levels of educational attainment for nonwhite children are the same regardless of whether their parents divorce. Among nonwhite children, parental divorce is one of many disadvantaged events faced during childhood, rendering the effect of any particular adverse event less adversely disruptive. Parental divorce may in fact offer some relief from family conflict and benefit children’s psychological wellbeing.

We assess the mechanisms explaining the impact of parental divorce on children’s education using a formal causal mediation analysis based on a counterfactual framework. This framework offers three key advantages. First, we test several key assumptions for estimating causal mediation effects. For example, we tested whether a linear model was sufficient to estimate mediating effects. We found that a parametric function was sufficient, but we note that our analytic approach could be applied to nonparametric scenarios as well. Second, we quantify the relative strength of mediating effects. Family income, and to a lesser extent family instability, provide the most robust explanation as to why divorce negatively impacts white children’s education. Despite the strong association between cognitive ability and educational outcomes, our results imply that any effect of divorce on cognitive skills does not translate into long-term educational inequality between children who grew up in divorced and two-parent families. Children’s psychosocial skills are more malleable throughout childhood, susceptible to the influence of family shocks, and important in linking disruption to lower levels of education than children’s cognitive skills.

Third, we provide a formal sensitivity analysis for total and mediating effects. Our statistical estimation of causal total and mediation effects requires the assumption of sequential ignorability. If this assumption holds true, we have obtained valid estimates of the total and mediating causal effects of parental divorce. Yet divorce is a highly selective process; we cannot plausibly account for all the time-varying factors that influence both parents’ likelihood of divorce and children’s educational outcomes and associated mediators. A researcher can begin with the ignorability assumption in order to carry out meaningful analyses without necessarily committing oneself to the validity of the assumption. We supplement our results with sensitivity analyses that show the extent to which the effect estimates remain valid when the ignorability assumption is violated. The sensitivity analyses indicate that the magnitude of any potential unobserved variable needs to be very large to alter our inferences about the total effects of divorce and the mediation effect of family income.

We set out to explore the role of family conditions and children’s skills in explaining the impact of parental divorce on children’s education. The analyses yielded compelling answers. The effect of parental divorce on white children’s education is explained by divorce-induced declines in family income, family stability, and children’s psychosocial skills. Cognitive skills do not explain lower levels of educational attainment among children of divorced parents. That is, when parents divorce, children’s cognitive ability does not deteriorate; their emotional wellbeing does. This decline in psychosocial skills, alongside economic strain and family instability, helps explain why white children’s educational attainment suffers following parental divorce. Although this finding is consistent with prior research, it had not been quantified using causal mediation methods and sensitivity analyses. Our results thus strengthen confidence in this key finding. We also find that parental divorce does not limit the educational attainment of nonwhite children because declines in economic resources and stability are offset by increases in child wellbeing. Our results suggest two implications for family and social policy. First, policies that aim to promote the education of children who are impacted by parental divorce should prioritize minimizing the economic strain. Second, policies that prioritize martial stability among nonwhite children oversimplify the range of adversities these children face that limit their educational attainment and overlook the possible benefits to their parents separating.

## Supplementary Material

Supplemental Material

## Figures and Tables

**Table 1:** Descriptive statistics of predivorce characteristics, mediators, and educational outcomes.

	Parents not divorced		Parents divorced		t- test	balance test
	Mean	SD	Mean	SD		
Family Background Factors						
Black (binary 0/1)	0.09	-	0.13	-	†	
Hispanic (binary 0/1)	0.09	-	0.09	-		
Born in United States (binary 0/1)	0.94	-	0.97	-	†	
Southern residence at age 14 (binary 0/1)	0.30	-	0.35	-	†	
Raised no religious preference (binary 0/1)	0.03	-	0.04	-	*	
Intact family at age 14 (binary 0/1)	0.78	-	0.67	-	†	
Absent father before age 14 (binary 0/1)	0.14	-	0.22	-	†	
Sibship size (continuous 0–19)	3.56	2.36	3.59	2.35		*
Parents’ household income (thousands of dollars) (continuous 0–75)	19.44	12.44	16.06	10.42	†	*
Socioeconomic Factors						
Highest education is completed high school (binary 0/1)	0.56	-	0.59	-		
Highest education is completed college or more (binary 0/1)	0.23	-	0.08	-	†	
Employed (binary 0/1)	0.54	-	0.50	-	†	
Employed at a private company (binary 0/1)	0.03	-	0.02	-		
Job offers flexible hours (binary 0/1)	0.48	-	0.45	-		
Delinquent activity (binary 0/1)	0.65	-	0.76	-	†	
Log household income (continuous 4–14)	10.25	1.10	9.82	1.20	†	
Household below poverty line (binary 0/1)	0.13	-	0.18	-	†	
Household received welfare/TANF (binary 0/1)	0.10	-	0.21	-	†	
Cognitive and Psychosocial Factors						
Rotter Locus of Control Scale (continuous 4–16)	8.45	2.45	8.85	2.41	†	
Pearlin Mastery Scale (continuous 9–28)	22.20	3.03	21.71	3.22	†	
Rosenberg Self-Esteem Scale (continuous 240–650)	482.45	80.28	465.79	82.05	†	
Juvenile delinquent activity (binary 0/1)	0.93	-	0.94	-	*	
CESD score (continuous 0–21)	3.93	3.63	5.15	4.35	†	
Body mass index (continuous 11–42)	21.74	3.14	21.74	3.39		
Cognitive ability ASVAB (continuous −3 to 3)	−0.04	0.68	−0.19	0.63	†	*
High school class rank percentile (continuous 0–1)	0.41	0.22	0.48	0.20	†	
High school program was college preparatory (binary 0/1)	0.33	-	0.21	-	†	
Family Formation and Wellbeing Factors						
Sexual debut at age 15 or younger (binary 0/1)	0.11	-	0.17	-	†	
“Wife with family has no time for employment” (binary 0/1)	0.17	-	0.19	-		
Age at time of child’s birth (continuous 13–37)	26.27	4.45	24.12	4.63	†	
Previously married (binary 0/1)	0.09	-	0.12	-	†	
Log months between marriage and first birth (continuous 0–5)	2.75	1.33	2.53	1.31	†	†
Desired birth (continuous 0–13)^*a*^	1.13	1.31	0.98	1.40	†	
Undesired birth (continuous 0–8)^*a*^	0.24	0.60	0.32	0.71	†	
Child male (0/1)	0.53	-	0.51	-		
Child birth weight (ounces; continuous 6–268)	120.13	20.09	117.65	20.12	†	
Mother/father argue about chores often/ very often (binary 0/1)	0.19	-	0.14	-	†	
Mother/father argue about money often/ very often (binary 0/1)	0.21	-	0.09	-	†	
Mother/father argue about cheating often/ very often (binary 0/1)	0.08	-	0.07	-	†	
Mother/father argue about religion often/ very often (binary 0/1)	0.03	-	0.02	-		
Mother/father different race (binary 0/1)	0.09	-	0.13	-	†	
Mother/father raised different religious preference (binary 0/1)	0.46	-	0.41	-	†	
Mother/father difference in college completion (binary 0/1) Mediators Family Conditions	0.01		0.05		†	
*Mediators*						
Family Conditions						
Family income (continuous)	75,753	72,336	39,348	41,143	†	
Relationship transitions (continuous)	1.29	1.08	2.60	1.61	†	
Children’s Skills						
Psychosocial skills scale (continuous)	0.59	0.13	0.55	0.14	†	
Cognitive skills scale (continuous)	0.57	0.16	0.54	0.14	†	
*Outcomes*						
Children’s Educational Attainment						
High school completion (by age 18; binary 0/1)	0.85	0.35	0.76	0.43	†	
College attendance (by age 19; binary 0/1)^	0.62	0.48	0.46	0.50	†	
College completion (by age 23; binary 0/1)^	0.30	0.46	0.14	0.35	†	
Weighted sample proportion	0.66		0.33			
*N*	4,838		2,420			

*Notes:* Sample restricted to children who were at least 18 years old in 2012 and whose parents were married at the time of their birth. Parental divorce is measured as divorce that occurred when children were 0 to 17 years old. Factors refer to mothers unless otherwise specified. All factors are measured prior to the divorce interval (i.e., at the time of child’s birth or earlier). All descriptive statistics are weighted by the NLSY sample weight.

a “Desired birth” is the extent to which a mother’s 1979 fertility preference meets or exceeds a child’s birth order. “Undesired birth” is the extent to which a child’s birth order exceeds the mother’s 1979 fertility preference. Each measure equals zero when the measure does not go in the stated direction. The balance tests indicate whether the covariate remains a significant predictor of divorce in a model with the propensity of divorce included. TANF, Temporary Assistance for Needy Families.

* *p* ≤ 0.05

† *p* ≤ 0.01 (two-tailed tests).

**Table 2:** Total effects of parental divorce on children’s educational attainment by race.

	Total effects		
	Full sample	Whites	Nonwhites
Educational Attainment Outcomes			
High school completion	−0.04^†^	−0.07^†^	−0.01
	(0.01)	(0.02)	(0.02)
College attendance	−0.07^†^	−0.13^†^	0.00
	(0.02)	(0.02)	(0.02)
College completion	−0.07^†^	−0.13^†^	0.00
	(0.01)	(0.02)	(0.02)

*Notes*: Numbers in parentheses are standard errors. Sample is restricted to children who were at least 18 years old in 2012 and whose parents were married at the time of their birth. Parental divorce is measured as divorce that occurred when children were 0 to 17 years old. Estimates are based on linear probability models. Adjusted models control for propensity of parental divorce and children’s age in 2012 (estimates not shown). Propensity scores were estimated by a logit regression model of parental divorce on the set of predivorce covariates. Analytic sample (*N* = 5,176) is further restricted to ages 19 and older for college attendance (*N* = 4,982) and ages 23 and older for college completion (*N* = 3,901).

† *p* ≤ 0.01 (two-tailed tests).

**Table 3:** Total effects of parental divorce on children’s educational attainment by race and event age.

	Total effects	
	Whites	Nonwhites
Educational Attainment Outcomes		
Divorce Age 0–5		
High school completion	−0.069^†^	0.030
	(0.022)	(0.025)
College attendance	−0.122^†^	0.042
	(0.026)	(0.030)
College completion	−0.086^†^	0.039
	(0.022)	(0.022)
Divorce Age 6–11		
High school completion	−0.064^†^	−0.031
	(0.025)	(0.027)
College attendance	−0.122^†^	−0.044
	(0.031)	(0.031)
College completion	−0.116^†^	−0.008
	(0.026)	(0.021)
Divorce Age 12–17		
High school completion	−0.051	−0.023
	(0.030)	(0.034)
College attendance	−0.065	0.018
	(0.039)	(0.039)
College completion	−0.097^†^	−0.023
	(0.034)	(0.025)

*Notes*: Numbers in parentheses are standard errors. Sample is restricted to children whose parents were married at the time of their birth and who were at least 18 years old in 2012. Estimates are based on linear probability models. Adjusted models control for propensity of parental divorce and children’s age in 2012 (estimates not shown). Propensity scores were estimated by a logit regression model of parental divorce on the set of time-varying predivorce covariates. Analytic sample (*N* = 5,176) is further restricted to ages 19 and older for college attendance (*N* = 4,982) and ages 23 and older for college completion (*N* = 3,901).

† *p* < 0.01 (two-tailed tests).

**Table 4:** Mediation effects of parental divorce on children’s educational attainment: whites by event age.

	Divorce Age 0–5		Divorce Age 6–11		Divorce Age 12–17	
	Mediation effects	% mediated	Mediation effects	% mediated	Mediation effects	% mediated

Mediators of Effects of Divorce on High School Completion						
Family Conditions						
Family income	−0.02^†^	30%	−0.03^†^	41%	−0.05^†^	-
	(0.00)		(0.01)		(0.01)	
Relationship transitions	−0.02^†^	30%	−0.02*	28%	−0.03^†^	-
	(0.01)		(0.01)		(0.01)	
Children’s Skills						
Psychosocial skills scale	−0.02^†^	20%	0.00	-	−0.01*	-
	(0.00)		(0.00)		(0.00)	
Cognitive skills scale	0.00	-	0.00	-	0.00	-
	(0.00)		(0.01)		(0.01)	
Mediators of Effects of Divorce on College Attendance						
Family Conditions						
Family income	−0.03^†^	30%	−0.05^†^	37%	−0.08^†^	-
	(0.01)		(0.01)		(0.01)	
Relationship transitions	−0.03^†^	32%	−0.02	-	−0.02	-
	(0.01)		(0.01)		(0.01)	
Children’s Skills						
Psychosocial skills scale	−0.02^†^	21%	−0.01	-	−0.01*	-
	(0.01)		(0.01)		(0.01)	
Cognitive skills scale	0.00	-	0.00	-	0.01	-
	(0.01)		(0.01)		(0.01)	
Mediators of Effects of Divorce on College Completion						
Family Conditions						
Family income	−0.03^†^	32%	−0.04^†^	34%	−0.07^†^	67%
	(0.01)		(0.01)		(0.01)	
Relationship transitions	−0.04^†^	44%	−0.03*	26%	−0.03*	25%
	(0.01)		(0.01)		(0.01)	
Children’s Skills						
Psychosocial skills scale	−0.02^†^	25%	−0.01	-	−0.01*	15%
	(0.00)		(0.01)		(0.01)	
Cognitive skills scale	0.00	-	0.00	-	0.00	-
	(0.01)		(0.01)		(0.01)	

*Notes*: Numbers in parentheses are standard errors. Sample is restricted to children whose parents were married at the time of their birth and who were at least 18 years old in 2012. Estimates are based on linear probability models. All models control for propensity of parental divorce and children’s age in 2012 (estimates not shown). Propensity scores were estimated by a logit regression model of parental divorce on the set of time-invariant and time-varying predivorce covariates. Proportion mediated is only reported when the total effect and the indirect effect are both significant. Sample (*N* = 5,176) is further restricted to ages 19 and older for college attendance (*N* = 4,982) and to ages 23 and older for college completion (*N* = 3,901).

* *p* ≤ 0.05

† *p* < 0.01 (two-tailed tests).

**Table 5:** Mediation effects of parental divorce on children’s educational attainment: nonwhites by event age.

	Divorce Age 0–5		Divorce Age 6–11		Divorce Age 12–17	
	Mediation effects	% mediated	Mediation effects	% mediated	Mediation effects	% mediated
Mediators of Effects of Divorce on High School Completion						
Family Conditions						
Family income	0.001	–	−0.013	–	−0.059^†^	–
	(0.007)		(0.008)		(0.011)	
Relationship transitions	−0.020^†^	–	−0.020^†^	–	−0.013^†^	–
	(0.006)		(0.007)		(0.004)	
Children’s Skills						
Psychosocial skills scale	0.010	–	−0.004	–	0.001	–
	(0.006)		(0.006)		(0.008)	
Cognitive skills scale	0.028^†^	–	0.012	–	0.027*	–
	(0.008)		(0.009)		(0.012)	
Mediators of Effects of Divorce on College Attendance						
Family Conditions						
Family income	0.001	–	−0.013	–	−0.060^†^	–
	(0.007)		(0.008)		(0.011)	
Relationship transitions	−0.008	–	−0.014	–	−0.006	–
	(0.007)		(0.008)		(0.005)	
Children’s Skills						
Psychosocial skills scale	0.010	–	−0.004	–	0.001	–
	(0.006)		(0.006)		(0.007)	
Cognitive skills scale	0.041^†^	–	0.015	–	0.037*	–
	(0.011)		(0.011)		(0.015)	
Mediators of Effects of Divorce on College Completion						
Family Conditions						
Family income	0.001	–	−0.006	–	−0.025^†^	–
	(0.004)		(0.003)		(0.006)	
Relationship transitions	−0.005	–	−0.010	–	−0.004	–
	(0.005)		(0.006)		(0.003)	
Children’s Skills						
Psychosocial skills scale	0.006	–	−0.002	–	0.000	–
	(0.004)		(0.003)		(0.005)	
Cognitive skills scale	0.024^†^	–	0.009	–	0.022*	–
	(0.007)		(0.007)		(0.009)	

*Notes*: Numbers in parentheses are standard errors. Sample is restricted to children whose parents were married at the time of their birth and who were at least 18 years old in 2012. Estimates are based on linear probability models. All models control for propensity of parental divorce and children’s age in 2012 (estimates not shown). Propensity scores were estimated by a logit regression model of parental divorce on the set of time-invariant and time-varying predivorce covariates. Proportion mediated is only reported when the total effect and the indirect effect are both significant. Sample (*N* = 5,176) is further restricted to ages 19 and older for college attendance (*N* = 4,982) and to ages 23 and older for college completion (*N* = 3,901).

* *p* ≤ 0.05

† *p* ≤ 0.01 (two-tailed tests).

## Notes

1 Family disruption includes several possible forms of change in family structure, with the main line of demarcation being between adding and losing a partner. The loss of a partner is generally more negatively disruptive than the addition of a partner. Another line of demarcation lies between divorce among married parents versus union dissolution among cohabitating parents. The proportion of children born to cohabiting parents has increased over the last several decades (Kennedy and Bumpass 2008), yet such children continue to be more disadvantaged relative to children born to marital unions (Osborne and McLanahan 2007). If cohabitating unions are more disadvantaged and unstable from the onset than marital unions, the effects of dissolution on children may be less severe (Brown 2006; McLanahan et al. 2013). Although we considered assessing loss of a cohabitating partner, adding these cases to form a broader category of family disruption would have increased the heterogeneity of the effect estimates, and there were too few cases of those who began in cohabitating unions at the time of childbirth to allow for meaningful stratified estimates (particularly among subgroups).

2 Another plausible factor is fathers’ financial contributions to college. A portion of this factor may be captured by postdivorce family income. However, if fathers contribute directly to college, such effects would remain unaccounted for. We do not have data to capture this factor, particularly data to capture the intention of fathers to help finance college. That is, we would need to measure the intention to contribute if we were to consider it a mechanism for the population who do not attend college.

3 There is some variation according to which measures of psychosocial skills are consid¬ered (Aughinbaugh et al. 2005; Cherlin et al. 1991; D’Onofrio et al. 2007; Sun 2001; Sun and Li 2002).

4 A critique of the linear probability model (LPM) is that it does not estimate the structural parameters of a nonlinear model. However, the marginal effects, rather than structural parameters of a binary choice model, are our primary concern. The LPM performs reasonably well with respect to estimating the marginal effects. The LPM will not give the true marginal effects from the nonlinear model, yet neither will an incorrect nonlinear model (Angrist and Pischke 2009). The LPM is particularly useful when we interpret mediation effects in terms of probabilities rather than odds ratios.

5 We can also define the direct effect (DE) for unit *i* as follows: *DE_i_* = *Y_i_* (1, *M_i_* (*d*)) − *Y_i_* (0, *M_i_* (*d*)). The direct effect represents the expected difference in education as a result of all possible mechanisms other than the one under consideration by deactivating the pathway of the mediator under consideration. In the absence of interactions with the treatment, the total effect is equivalent to the sum of the indirect and direct effects.

6 The causal mediation effect obtained at *D* = 1 may be different from that at *D* = 0 if there is an interaction between the treatment and mediator variables. We did not find significant interactions for most mediators and thus do not include them in the outcome models.

7 The bias term in the sensitivity analyses for the mediation effects is *B* = −*γλ*, where the bias factors refer to *γ* = *E*(*Y*|*U* = 1, *D = d, M = m, P = p*)−*E*(*Y|U* = 0, *D = d, M = m, P = p*) and *λ* = *P* (*U* = 1| *D* = 1, *P = p*)−*P*(*U* = 0|*D* = 0, *P = p*), respectively.

8 As a pseudo measure of whether a mother desired a child’s birth, we include a variable indicating the difference between a woman’s desired number of children and the birth order position of the current child. “Desired birth” is defined as children for whom the birth order position is less than the mother’s 1979 fertility preference. “Undesired birth” is defined as children for whom birth order position exceeds fertility preference. For example, if a mother desired three children, and the child is the fifth, the indicator for desired births takes a value of zero, and the indicator for undesired births takes a value of two.

9 A limitation of these data is that the majority of our covariates are based on mothers; the strength is that we have a large nationally representative sample with rich longitudinal data on mothers and their children. We also have enough data on fathers and households to construct several key sociodemographic characteristics and indicators of relationship quality.

10 The Pearlin Mastery Scale, a self-concept measure, indicates the extent to which individ¬uals perceive themselves to be in control of forces that significantly impact their lives. The Rosenberg Self-Esteem Scale measures the self-evaluation that an individual makes and customarily maintains. The TIPI is a 10-question instrument that yields five scales that measure the “Big Five” personality traits (i.e., extraversion, agreeableness, consci¬entiousness, emotional stability, and openness to experiences). Here, we focus on the Emotional Stability Scale. The BPI measures the frequency, range, and type of childhood behavior problems for children. This includes measures for antisocial behavior, anxious or depressive behavior, conflict behavior, and dependent behavior. Finally, the CESD is a self-report scale that measures the prevalence of depression symptoms. Respondents were asked whether they never/rarely, sometimes, occasionally, or most/all of the time (1) had poor appetite, (2) had trouble keeping their mind on tasks, (3) were depressed, (4) felt that everything took extra effort, (5) had restless sleep, (6) were sad, and (7) could not get going.

11 We explored each of the indicators separately for both cognitive and psychosocial skills but ultimately decided that summary scales resulted in little loss of information and greatly increased parsimony.

12 If we had instead compared postdivorce mediator measures of the treated group to mediator measures of the control group across childhood, we would expect bias in our estimates due to differences in the child’s mean age at the time of mediator measurement between the divorce and nondivorce groups.

13 Missing mediator values were imputed with a model based on covariates included in Table 1. Models that use imputed and nonimputed mediating variables produce substantively similar results.

14 In additional analyses (not shown), we use a variety of machine learning techniques (i.e., classification and regression trees and ensemble methods) to estimate the likelihood of parental divorce. The correlation with the simple logit specification is high, and we use this for simplicity.

15 In additional sensitivity analyses (not shown), we also explore *γ* ranging from −40 to 40. Our conclusions remain very similar. The results are available upon request.
