# Supplementary material for: Why Does Parental Divorce Lower Children’s Educational Attainment? A Causal Mediation Analysis
Source: Sociol Sci. Author manuscript; Available in PMC 2019 Jun 11. (PMC6559749; doi:10.15195/v6.a11)
Supplement: Supplemental Material [file NIHMS1025320-supplement-Supplemental_Material.pdf]

sociological science

Supplement to:

Brand, Jennie E., Ravaris Moore, Xi Song, and Yu Xie. 2019. “Why Does Parental Divorce Lower Children’s Educational Attainment? A Causal Mediation Analysis.” Sociological Science 6: 264-292.

Appendix Table A
Regression Estimates Predicting Child Experiencing Parental Divorce

	β/(SE)	
**Family Background Factors**		
Black (binary 0/1)	0.04	
	(0.09)	
Hispanic (binary 0/1)	-0.03	
	(0.08)	
U.S. born (binary 0/1)	0.47	†
	(0.12)	
Southern residence at age 14 (binary 0/1)	0.13	*
	(0.06)	
Raised no religious preference (binary 0/1)	-0.06	
	(0.15)	
Two-parent family at age 14 (binary 0/1)	-0.11	
	(0.07)	
Absent father before age 14 (binary 0/1)	0.20	*
	(0.08)	
Sibship size (continuous 0-19)	-0.06	†
	(0.01)	
Parents' household income ($1,000s) (continuous 0-75)	0.00	
	(0.00)	
**Socioeconomic Factors**		
Highest education is completed high school (binary 0/1)	-0.16	*
	(0.08)	
Highest education is completed college or more (binary 0/1)	-0.90	†
	(0.14)	
Employed (binary 0/1)	0.32	†
	(0.10)	
Employed at a private company (binary 0/1)	0.36	
	(0.21)	
Job offers flexible hours (binary 0/1)	-0.20	*
	(0.10)	
Delinquent activity (binary 0/1)	0.23	†
	(0.06)	
Log household income (continuous 4-14)	-0.06	*
	(0.03)	
Household below poverty line (binary 0/1)	-0.09	
	(0.07)	
Household received welfare/TANF (binary 0/1)	0.42	†
	(0.07)	
**Cognitive and Psychosocial Factors**		
Rotter Locus of Control scale (continuous 4-16)	0.01	
	(0.01)	
Pearlin Mastery scale (continuous 9-28)	0.03	*
	(0.01)	
Rosenberg Self-Esteem scale (continuous 240-650)	0.00	
	(0.00)	
Juvenile delinquent activity (binary 0/1)	0.06	
	(0.11)	

CESD score (continuous 0-21)	0.05	†
	(0.01)	
Body mass index (continuous 11-42)	-0.03	†
	(0.01)	
Cognitive ability ASVAB (continuous -3-3)	0.14	*
	(0.05)	
High school class rank percentile (continuous 0-1)	0.34	*
	(0.16)	
High school program was college prep (binary 0/1)	-0.06	
	(0.07)	
**Family Formation and Wellbeing Factors**		
Sexual debut at age 15 or younger (binary 0/1)	0.19	*
	(0.08)	
"Wife with family has no time for employment" (binary 0/1)	-0.17	*
	(0.07)	
Age at time of child's birth (continuous 13-37)	-0.09	†
	(0.01)	
Previously married (binary 0/1)	0.46	†
	(0.09)	
Log months between marriage and first birth (continuous 0-5)	0.19	†
	(0.02)	
Desired birth (continuous 0-13)	-0.07	†
	(0.02)	
Undesired birth (continuous 0-8)	0.13	†
	(0.04)	
Child male (0/1)	-0.07	
	(0.05)	
Child birth weight (ounces; continuous 6-268)	0.00	*
	(0.00)	
Mother/father argue about chores often/very often (binary 0/1)	0.22	*
	(0.11)	
Mother/father argue about money often/very often (binary 0/1)	-0.41	†
	(0.11)	
Mother/father argue about cheating often/very often (binary 0/1)	0.11	
	(0.14)	
Mother/father argue about religion often/very often (binary 0/1)	-0.15	
	(0.21)	
Mother/father different race (binary 0/1)	0.20	*
	(0.10)	
Mother/father raised different religious preference (binary 0/1)	-0.20	†
	(0.06)	
Mother/father difference in college completion (binary 0/1)	2.26	†
	(0.25)	
Intercept	1.85	†
	(0.50)	
N	7258	
Log Likelihood	-4114.30	
$P > \chi^2$	0	

Notes: Numbers in parentheses are standard errors. Sample restricted to children who were 18 years old in 2012 and whose parents were married at the time of their birth. Parental divorce is measured as divorce that occurred when children were 0-17 years old. Factors refer to mothers unless otherwise specified. All factors are measured prior to the divorce interval, i.e. at the time of child's birth or earlier.
* $p \le 0.05$ † $p \le 0.001$; two-tailed tests;

Appendix Table B1
Sensitivity Results for Total Effects of Parental Divorce on Children's Educational Attainment: Whites, by Event Age

	Sensitivity parameters		Divorce Age 0-5		Divorce Age 6-11		Divorce Age 12-17	
**Educational Attainment**	γ	λ	Total effects	CI	Total effects	CI	Total effects	CI
High School Completion	-40%	-10%	-0.11	(-0.15, -0.07)	-0.10	(-0.15, -0.06)	-0.09	(-0.15, -0.03)
	-20%	-10%	-0.09	(-0.13, -0.05)	-0.08	(-0.13, -0.04)	-0.07	(-0.13, -0.01)
	-10%	-10%	-0.08	(-0.12, -0.04)	-0.07	(-0.12, -0.03)	-0.06	(-0.12, 0.00)
	-40%	-5%	-0.09	(-0.13, -0.05)	-0.08	(-0.13, -0.04)	-0.07	(-0.13, -0.01)
	-20%	-5%	-0.08	(-0.12, -0.04)	-0.07	(-0.12, -0.03)	-0.06	(-0.12, 0.00)
	-10%	-5%	-0.07	(-0.12, -0.03)	-0.07	(-0.12, -0.02)	-0.06	(-0.11, 0.00)
	10%	-5%	-0.06	(-0.11, -0.02)	-0.06	(-0.11, -0.01)	-0.05	(-0.10, 0.01)
	20%	-5%	-0.06	(-0.10, -0.02)	-0.05	(-0.10, -0.01)	-0.04	(-0.10, 0.02)
	40%	-5%	-0.05	(-0.09, -0.01)	-0.04	(-0.09, 0.00)	-0.03	(-0.09, 0.03)
	10%	-10%	-0.06	(-0.10, -0.02)	-0.05	(-0.10, -0.01)	-0.04	(-0.10, 0.02)
	20%	-10%	-0.05	(-0.09, -0.01)	-0.04	(-0.09, 0.00)	-0.03	(-0.09, 0.03)
	40%	-10%	-0.03	(-0.07, 0.01)	-0.02	(-0.07, 0.02)	-0.01	(-0.07, 0.05)
College Attendance	-40%	-10%	-0.16	(-0.21, -0.11)	-0.16	(-0.22, -0.10)	-0.11	(-0.18, -0.03)
	-20%	-10%	-0.14	(-0.19, -0.09)	-0.14	(-0.20, -0.08)	-0.09	(-0.16, -0.01)
	-10%	-10%	-0.13	(-0.18, -0.08)	-0.13	(-0.19, -0.07)	-0.08	(-0.15, 0.00)
	-40%	-5%	-0.14	(-0.19, -0.09)	-0.14	(-0.20, -0.08)	-0.09	(-0.16, -0.01)
	-20%	-5%	-0.13	(-0.18, -0.08)	-0.13	(-0.19, -0.07)	-0.08	(-0.15, 0.00)
	-10%	-5%	-0.13	(-0.18, -0.08)	-0.13	(-0.19, -0.07)	-0.07	(-0.15, 0.01)
	10%	-5%	-0.12	(-0.17, -0.07)	-0.12	(-0.18, -0.06)	-0.06	(-0.14, 0.02)
	20%	-5%	-0.11	(-0.16, -0.06)	-0.11	(-0.17, -0.05)	-0.06	(-0.13, 0.02)
	40%	-5%	-0.10	(-0.15, -0.05)	-0.10	(-0.16, -0.04)	-0.05	(-0.12, 0.03)
	10%	-10%	-0.11	(-0.16, -0.06)	-0.11	(-0.17, -0.05)	-0.06	(-0.13, 0.02)
	20%	-10%	-0.10	(-0.15, -0.05)	-0.10	(-0.16, -0.04)	-0.05	(-0.12, 0.03)
	40%	-10%	-0.08	(-0.13, -0.03)	-0.08	(-0.14, -0.02)	-0.03	(-0.10, 0.05)
College Completion	-40%	-10%	-0.13	(-0.17, -0.08)	-0.16	(-0.21, -0.11)	-0.14	(-0.20, -0.07)
	-20%	-10%	-0.11	(-0.15, -0.06)	-0.14	(-0.19, -0.09)	-0.12	(-0.18, -0.05)
	-10%	-10%	-0.10	(-0.14, -0.05)	-0.13	(-0.18, -0.08)	-0.11	(-0.17, -0.04)
	-40%	-5%	-0.11	(-0.15, -0.06)	-0.14	(-0.19, -0.09)	-0.12	(-0.18, -0.05)
	-20%	-5%	-0.10	(-0.14, -0.05)	-0.13	(-0.18, -0.08)	-0.11	(-0.17, -0.04)
	-10%	-5%	-0.09	(-0.13, -0.05)	-0.12	(-0.17, -0.07)	-0.10	(-0.17, -0.04)
	10%	-5%	-0.08	(-0.12, -0.04)	-0.11	(-0.16, -0.06)	-0.09	(-0.16, -0.03)
	20%	-5%	-0.08	(-0.12, -0.03)	-0.11	(-0.16, -0.06)	-0.09	(-0.15, -0.02)
	40%	-5%	-0.07	(-0.11, -0.02)	-0.10	(-0.15, -0.05)	-0.08	(-0.14, -0.01)
	10%	-10%	-0.08	(-0.12, -0.03)	-0.11	(-0.16, -0.06)	-0.09	(-0.15, -0.02)
	20%	-10%	-0.07	(-0.11, -0.02)	-0.10	(-0.15, -0.05)	-0.08	(-0.14, -0.01)
	40%	-10%	-0.05	(-0.09, 0.00)	-0.08	(-0.13, -0.03)	-0.06	(-0.12, 0.01)

Notes: γ_S refers to the mean difference in children's education associated with a unit difference in the binary unobserved confounder conditional on divorce status and propensity scores. λ_S refers to the prevalence difference of the binary unobserved confounder between the children of divorced and non-divorced parents conditional on propensity scores. The bias factor is equal to the product of the two parameters, and we subtract this bias factor from the total effect and the confidence interval.

Appendix Table B2
Sensitivity Results for Mediation Effects of Parental Divorce on Children's Educational Attainment: Whites, by Event Age

	Sensitivity parameters		Divorce Age 0-5		Divorce Age 6-11		Divorce Age 12-17	
**Educational Attainment**	γ	λ	Med. effects	CI	Med. effects	CI	Med. effects	CI
High School Completion								
**Family Conditions**								
Family income	-20%	-10%	-0.04	(-0.05, -0.03)	-0.05	(-0.06, -0.04)	-0.07	(-0.09, -0.05)
	-10%	-10%	-0.03	(-0.04, -0.02)	-0.04	(-0.05, -0.03)	-0.06	(-0.08, -0.04)
	-20%	-5%	-0.03	(-0.04, -0.02)	-0.04	(-0.05, -0.03)	-0.06	(-0.08, -0.04)
	-10%	-5%	-0.03	(-0.04, -0.02)	-0.04	(-0.05, -0.03)	-0.06	(-0.07, -0.04)
	10%	-5%	-0.02	(-0.03, -0.01)	-0.03	(-0.04, -0.02)	-0.05	(-0.06, -0.03)
	20%	-5%	-0.01	(-0.02, 0.00)	-0.02	(-0.03, -0.01)	-0.04	(-0.06, -0.02)
	10%	-10%	-0.01	(-0.02, 0.00)	-0.02	(-0.03, -0.01)	-0.04	(-0.06, -0.02)
	20%	-10%	0.00	(-0.01, 0.01)	-0.01	(-0.02, 0.00)	-0.03	(-0.05, -0.01)
Relationship transitions	-20%	-10%	-0.04	(-0.06, -0.03)	-0.04	(-0.06, -0.02)	-0.05	(-0.06, -0.03)
	-10%	-10%	-0.03	(-0.05, -0.02)	-0.03	(-0.05, -0.01)	-0.04	(-0.05, -0.02)
	-20%	-5%	-0.03	(-0.05, -0.02)	-0.03	(-0.05, -0.01)	-0.04	(-0.05, -0.02)
	-10%	-5%	-0.03	(-0.05, -0.01)	-0.03	(-0.05, -0.01)	-0.03	(-0.05, -0.01)
	10%	-5%	-0.02	(-0.04, 0.00)	-0.02	(-0.04, 0.00)	-0.02	(-0.04, 0.00)
	20%	-5%	-0.01	(-0.03, 0.00)	-0.01	(-0.03, 0.01)	-0.02	(-0.03, 0.00)
	10%	-10%	-0.01	(-0.03, 0.00)	-0.01	(-0.03, 0.01)	-0.02	(-0.03, 0.00)
	20%	-10%	0.00	(-0.02, 0.01)	0.00	(-0.02, 0.02)	-0.01	(-0.02, 0.01)
**Children's Skills**								
Psychosocial skills	-20%	-10%	-0.04	(-0.04, -0.03)	-0.02	(-0.03, -0.02)	-0.03	(-0.04, -0.02)
	-10%	-10%	-0.03	(-0.03, -0.02)	-0.01	(-0.02, -0.01)	-0.02	(-0.03, -0.01)
	-20%	-5%	-0.03	(-0.03, -0.02)	-0.01	(-0.02, -0.01)	-0.02	(-0.03, -0.01)
	-10%	-5%	-0.02	(-0.03, -0.01)	-0.01	(-0.02, 0.00)	-0.01	(-0.02, -0.01)
	10%	-5%	-0.01	(-0.02, 0.00)	0.00	(-0.01, 0.01)	0.00	(-0.01, 0.00)
	20%	-5%	-0.01	(-0.01, 0.00)	0.01	(0.00, 0.01)	0.00	(-0.01, 0.01)
	10%	-10%	-0.01	(-0.01, 0.00)	0.01	(0.00, 0.01)	0.00	(-0.01, 0.01)
	20%	-10%	0.00	(0.00, 0.01)	0.02	(0.01, 0.02)	0.01	(0.00, 0.02)
College Attendance								
**Family Conditions**								
Family income	-20%	-10%	-0.05	(-0.07, -0.04)	-0.07	(-0.08, -0.05)	-0.10	(-0.12, -0.08)
	-10%	-10%	-0.04	(-0.06, -0.03)	-0.06	(-0.07, -0.04)	-0.09	(-0.11, -0.07)
	-20%	-5%	-0.04	(-0.06, -0.03)	-0.06	(-0.07, -0.04)	-0.09	(-0.11, -0.07)
	-10%	-5%	-0.04	(-0.05, -0.03)	-0.05	(-0.07, -0.04)	-0.09	(-0.11, -0.06)
	10%	-5%	-0.03	(-0.04, -0.02)	-0.04	(-0.06, -0.03)	-0.08	(-0.10, -0.05)
	20%	-5%	-0.02	(-0.04, -0.01)	-0.04	(-0.05, -0.02)	-0.07	(-0.09, -0.05)
	10%	-10%	-0.02	(-0.04, -0.01)	-0.04	(-0.05, -0.02)	-0.07	(-0.09, -0.05)
	20%	-10%	-0.01	(-0.03, 0.00)	-0.03	(-0.04, -0.01)	-0.06	(-0.08, -0.04)
Relationship transitions	-20%	-10%	-0.05	(-0.07, -0.03)	-0.04	(-0.07, -0.02)	-0.04	(-0.06, -0.02)
	-10%	-10%	-0.04	(-0.06, -0.02)	-0.03	(-0.06, -0.01)	-0.03	(-0.05, -0.01)

	-20%	-5%	-0.04	(-0.06, -0.02)	-0.03	(-0.06, -0.01)	-0.03	(-0.05, -0.01)
	-10%	-5%	-0.04	(-0.06, -0.02)	-0.03	(-0.05, 0.00)	-0.03	(-0.05, 0.00)
	10%	-5%	-0.03	(-0.05, -0.01)	-0.02	(-0.04, 0.01)	-0.02	(-0.04, 0.01)
	20%	-5%	-0.02	(-0.04, 0.00)	-0.01	(-0.04, 0.01)	-0.01	(-0.03, 0.01)
	10%	-10%	-0.02	(-0.04, 0.00)	-0.01	(-0.04, 0.01)	-0.01	(-0.03, 0.01)
	20%	-10%	-0.01	(-0.03, 0.01)	0.00	(-0.03, 0.02)	0.00	(-0.02, 0.02)
**Children's Skills**								
Psychosocial skills	-20%	-10%	-0.04	(-0.05, -0.03)	-0.03	(-0.04, -0.02)	-0.03	(-0.05, -0.02)
	-10%	-10%	-0.03	(-0.04, -0.02)	-0.02	(-0.03, -0.01)	-0.02	(-0.04, -0.01)
	-20%	-5%	-0.03	(-0.04, -0.02)	-0.02	(-0.03, -0.01)	-0.02	(-0.04, -0.01)
	-10%	-5%	-0.03	(-0.04, -0.02)	-0.01	(-0.02, 0.00)	-0.02	(-0.03, -0.01)
	10%	-5%	-0.02	(-0.03, -0.01)	0.00	(-0.01, 0.01)	-0.01	(-0.02, 0.00)
	20%	-5%	-0.01	(-0.02, 0.00)	0.00	(-0.01, 0.01)	0.00	(-0.02, 0.01)
	-20%	-10%	-0.04	(-0.05, -0.03)	-0.03	(-0.04, -0.02)	-0.03	(-0.05, -0.02)
	-10%	-10%	-0.03	(-0.04, -0.02)	-0.02	(-0.03, -0.01)	-0.02	(-0.04, -0.01)
College Completion								
**Family Conditions**								
Family income	-20%	-10%	-0.05	(-0.06, -0.04)	-0.06	(-0.07, -0.05)	-0.09	(-0.11, -0.06)
	-10%	-10%	-0.04	(-0.05, -0.03)	-0.05	(-0.06, -0.04)	-0.08	(-0.10, -0.05)
	-20%	-5%	-0.04	(-0.05, -0.03)	-0.05	(-0.06, -0.04)	-0.08	(-0.10, -0.05)
	-10%	-5%	-0.03	(-0.04, -0.02)	-0.04	(-0.06, -0.03)	-0.07	(-0.09, -0.05)
	10%	-5%	-0.02	(-0.03, -0.01)	-0.03	(-0.05, -0.02)	-0.06	(-0.08, -0.04)
	20%	-5%	-0.02	(-0.03, -0.01)	-0.03	(-0.04, -0.02)	-0.06	(-0.08, -0.03)
	10%	-10%	-0.02	(-0.03, -0.01)	-0.03	(-0.04, -0.02)	-0.06	(-0.08, -0.03)
	20%	-10%	-0.01	(-0.02, 0.00)	-0.02	(-0.03, -0.01)	-0.05	(-0.07, -0.02)
Relationship transitions	-20%	-10%	-0.06	(-0.08, -0.04)	-0.06	(-0.08, -0.04)	-0.09	(-0.11, -0.06)
	-10%	-10%	-0.05	(-0.07, -0.03)	-0.05	(-0.07, -0.03)	-0.08	(-0.10, -0.05)
	-20%	-5%	-0.05	(-0.07, -0.03)	-0.05	(-0.07, -0.03)	-0.08	(-0.10, -0.05)
	-10%	-5%	-0.04	(-0.06, -0.02)	-0.04	(-0.07, -0.02)	-0.07	(-0.09, -0.05)
	10%	-5%	-0.03	(-0.05, -0.01)	-0.03	(-0.06, -0.01)	-0.06	(-0.08, -0.04)
	20%	-5%	-0.03	(-0.05, -0.01)	-0.03	(-0.05, -0.01)	-0.06	(-0.08, -0.03)
	10%	-10%	-0.03	(-0.05, -0.01)	-0.03	(-0.05, -0.01)	-0.06	(-0.08, -0.03)
	20%	-10%	-0.02	(-0.04, 0.00)	-0.02	(-0.04, 0.00)	-0.05	(-0.07, -0.02)
**Children's Skills**								
Psychosocial skills	-20%	-10%	-0.04	(-0.05, -0.03)	-0.05	(-0.06, -0.04)	-0.03	(-0.05, -0.02)
	-10%	-10%	-0.03	(-0.04, -0.02)	-0.04	(-0.05, -0.03)	-0.02	(-0.04, -0.01)
	-20%	-5%	-0.03	(-0.04, -0.02)	-0.04	(-0.05, -0.03)	-0.02	(-0.04, -0.01)
	-10%	-5%	-0.03	(-0.04, -0.02)	-0.04	(-0.05, -0.02)	-0.02	(-0.03, -0.01)
	10%	-5%	-0.02	(-0.03, -0.01)	-0.03	(-0.04, -0.01)	-0.01	(-0.02, 0.00)
	20%	-5%	-0.01	(-0.02, 0.00)	-0.02	(-0.03, -0.01)	0.00	(-0.02, 0.01)
	10%	-10%	-0.01	(-0.02, 0.00)	-0.02	(-0.03, -0.01)	0.00	(-0.02, 0.01)
	20%	-10%	0.00	(-0.01, 0.01)	-0.01	(-0.02, 0.00)	0.01	(-0.01, 0.02)

Notes: γ_S refers to the mean difference in children's education associated with a unit difference in the binary unobserved confounder conditional on divorce status, propensity scores, and mediator values. λ_S refers to the prevalence difference of the binary unobserved confounder between children of divorced and non-divorced parents conditional on divorce status, propensity scores, and mediator values. The bias factor is equal to the negation of the product of the two parameters, and we subtract this bias factor from the mediation effect and the confidence interval.
